# End-stage renal disease and survival in people with diabetes: a national database linkage study

**DOI:** 10.1093/qjmed/hcu170

**Published:** 2014-08-19

**Authors:** S. Bell, E.H. Fletcher, I. Brady, H.C. Looker, D. Levin, N. Joss, J.P. Traynor, W. Metcalfe, B. Conway, S. Livingstone, G. Leese, S. Philip, S. Wild, N. Halbesma, N. Sattar, R.S. Lindsay, J. McKnight, D. Pearson, H.M. Colhoun

**Affiliations:** From the ^1^Renal Unit, Ninewells Hopsital, NHS Tayside, Dundee DD1 9SY, ^2^Diabetes Epidemiology Unit, University of Dundee, Mackenzie Building, Kirsty Semple Way, Dundee DD2 4BF, ^3^NHS Highland, Raigmore Hospital, Inverness IV2 3UJ, ^4^Scottish Renal Registry, Cirrus House, Marchburn Drive, Glasgow Airport Business Park, Abbotsinch Paisley PA3 2SJ, ^5^Centre for Cardiovascular Science, University of Edinburgh, The Queens Medical Research Institute, 47 Little France Crescent, Edinburgh EH16 4TJ, ^6^Scottish Diabetes Research Network, Ninewells Hospital and Medical School, Dundee DD1 9SY, ^7^Grampian Diabetes Research Unit, Woolmanhill Hospital, Aberdeen AB25 1LD, ^8^Centre for Population Health Sciences, University of Edinburgh, Teviot Place, Edinburgh EH8 9AG, ^9^BHF Glasgow Cardiovascular Research Centre, University of Glasgow, 126 University Place, Glasgow G12 8TA, ^10^Metabolic Unit, Western General Hospital, Crewe Road South, Edinburgh EH4 2XU and ^11^NHS Grampian, Aberdeen Royal Infirmary, Eday Road, Aberdeen AB15 6XS, Scotland

## Abstract

**Background:** Increasing prevalence of diabetes worldwide is projected to lead to an increase in patients with end-stage renal disease (ESRD) requiring renal replacement therapy (RRT).

**Aim:** To provide contemporary estimates of the prevalence of ESRD and requirement for RRT among people with diabetes in a nationwide study and to report associated survival.

**Methods:** Data were extracted and linked from three national databases: Scottish Renal Registry, Scottish Care Initiative-Diabetes Collaboration and National Records of Scotland death data. Survival analyses were modelled with Cox regression.

**Results:** Point prevalence of chronic kidney disease (CKD)5 in 2008 was 1.63% of 19 414 people with type 1 diabetes (T1DM) compared with 0.58% of 167 871 people with type 2 diabetes (T2DM) (odds ratio for DM type 0.97, *P* = 0.77, on adjustment for duration. Although 83% of those with T1DM and CKD5 and 61% of those with T2DM and CKD5 were receiving RRT, there was no difference when adjusted for age, sex and DM duration (odds ratio for DM type 0.83, *P* = 0.432). Diabetic nephropathy was the primary renal diagnosis in 91% of people with T1DM and 58% of people with T2DM on RRT. Median survival time from initiation of RRT was 3.84 years (95% CI 2.77, 4.62) in T1DM and 2.16 years (95% CI: 1.92, 2.38) in T2DM.

**Conclusion**: Considerable numbers of patients with diabetes continue to progress to CKD5 and RRT. Almost half of all RRT cases in T2DM are considered to be due to conditions other than diabetic nephropathy. Median survival time for people with diabetes from initiation of RRT remains poor. These prevalence data are important for future resource planning.

## Introduction

The prevalence of diabetes worldwide has increased dramatically in recent years and is projected to reach over 552 million cases by 2030.^1^ In Scotland, the recorded prevalence of diabetes has increased substantially from 2.6%^2^ in 2003 to 4.7%^3^ in 2011. This increase in prevalence of diabetes is projected to lead to a significant increase in patients with end-stage renal disease (ESRD) requiring renal replacement therapy (RRT).^4^ Incidence of new patients commencing RRT in 2011 in Scotland was 96 per million population (pmp) with 24% of patients with diabetes as their primary renal diagnosis (PRD) between 2007 and 2011.^5^ UK Registry incidence was 108 pmp with 25% diabetes as PRD in their 2011 incident cohort.^6^ In the UK renal registry comorbidity data are reported in 55% of all those with RRT and among these 35% either have diabetes as the PRD or report it as a comorbid condition.^7^ However, to predict this future burden on healthcare and resources and to gauge whether outcomes in those with diabetes are changing, accurate estimates of current prevalence among those with diabetes would be useful but this cannot be directly obtained from current reports.

The aim of this study was first to examine the prevalence of patients with chronic kidney disease (CKD)5 and patients receiving RRT in all diabetics within Scotland, UK and ascertain the PRD of these patients. Second, we examined survival in these patients and whether PRD was related to survival after the initiation of RRT.

## Methods

All patients diagnosed with diabetes until 31 May 2008 in Scotland, UK were included in the analysis. Data were linked between the following relevant datasets: Scottish Renal Registry (SRR),^5^ the Scottish Care Initiative-Diabetes Collaboration (SCI-DC)^8^ and the National Records of Scotland death data by the National Health Service Information Services Division. Records for all those ever registered as having diabetes in the SCI-DC database by 31 May 2008 were extracted and linked to the SRR and National Records of Scotland death data. Diabetes type was determined by the clinician recording the diagnosis on the SCI-DC database but with the additional requirement that the prescription history did not contradict this (i.e. no evidence of lengthy period of diabetes before insulin and no co-prescribing of nonmetformin oral diabetes drugs.^9^

### Data sources

All patients with diabetes are registered on a single Nationwide Clinical Record System; the SCI-DC database which was rolled out nationwide from 2002 onwards. Registration occurs automatically when a patient is diagnosed with diabetes and assigned a corresponding Read code for diabetes in a primary care practice or a hospital-based diabetes clinic. Read codes are standardized codes used to record clinical data in primary care in the UK and are used to evaluate clinical performance, inform payment tariffs in primary care and allow inclusion into the national retinopathy screening programme. All but five of ∼1000 general practitioner practices in Scotland are linked to the register and so the database is estimated to capture over 99% of all patients nationally assigned a read code for diabetes.

The SRR records the assigned PRD, methods of treatment and outcomes of all patients receiving RRT in Scotland. Patients are registered when RRT first commenced. The SRR was started in 1991 and was back filled to 1960 when regular and routine RRT was initiated, using the European Renal Association—European Dialysis and Transplant Association (ERA-EDTA) registry. The SRR currently captures data from all of the nine adult and one paediatric renal unit in Scotland in addition to all 24 satellite dialysis units, thereby achieving 100% national coverage.

The PRD is recorded by the nephrologist responsible for the care of the patient using ERA-EDTA PRD codes. PRDs were grouped into five categories—primary glomerulonephritis, interstitial nephropathies, multisystem disease, diabetic nephropathy and ‘not known and other’.^10^ The not known and other group constitutes mostly those patients for whom it has not been possible to determine the PRD. A PRD of diabetic nephropathy is often based on the clinical judgement of the nephrologist and not proven by a renal biopsy. Renal biopsy is only performed when there is clinical doubt about the diagnosis of diabetic nephropathy due to the potential risks involved.

### Point prevalence

Data from individuals who were alive and diagnosed with diabetes on or before 31 May 2008 were analysed to provide the point prevalence in order to allow examination of the current level of renal disease burden. Individuals were identified as having CKD5 (estimated glomerular filtration rate [eGFR] < 15 ml/min/1.73 m^2^ or on RRT) using either modification of diet in renal disease^11^ derived eGFR readings obtained from the SCI-DC database or if they were receiving RRT on the 31 May 2008 as determined from the SRR. Only individuals with a minimum of two eGFR readings of <15 ml/min/1.73 m^2^ occurring at least 3 months apart in the 2 years prior to 31 May 2008 or in the 6 months after were included to minimize the risk of misclassifying acute kidney injury.

### Survival

An incident cohort of RRT recipients was selected to examine whether survival varied by PRD. This comprises those individuals in the diabetes SRR database extract who had first received RRT between 1 January 2006 and 13 January 2011. Records were linked to National Records of Scotland death data allowing mortality follow-up to the 13 January 2011.

### Statistical methods

Univariate tests of differences in the proportion of those with diabetes who were receiving RRT by diabetes type and by sex were carried out using Chi-squared tests with subsequent adjustment for possible confounders using logistic regression models. Cox regression survival analysis was used to determine whether survival from initiation of RRT varied by assigned PRD. All analyses were performed using R version 2.15.1.

### Ethics statement

Approval for data linkage was obtained from the Scotland A Research Ethics Committee, Caldicott (data privacy) Guardian for the 14 Scottish Health Boards and the National Health Service Information Services Division Privacy Advisory Committee.

## Results

**Figure 1.**
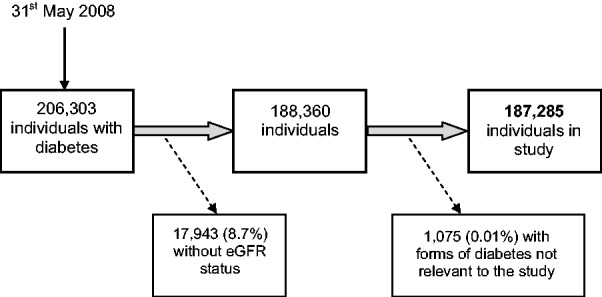
Identification of records included in study.

A total of 206 303 individuals were identified as having diabetes and being alive on the 31 May 2008 (Figure 1). A total of 18 091 (8.8%) did not have sufficient eGFR measurements to determine eGFR status. Of these, 148 were receiving RRT and so were included in the analysis (the remaining 17 943 individuals were excluded). In those with sufficient eGFR measurement, the median number of eGFR readings recorded was 5 (interquartile range 3–8) between the 2 years prior to 31 May 2008 up to 6 months after. A further 1075 individuals were also excluded as they had other forms of diabetes not relevant to this study.

### Point prevalence of CKD5

On 31 May 2008, there were 187 285 people in Scotland with known eGFR status registered as having type 1 diabetes (T1DM) or type 2 diabetes (T2DM). A total of 1288 of these had CKD5. Overall, 1.63% of those with T1DM had CKD5 compared with 0.58% of those with T2DM. The odds ratio for CKD5 in T1DM vs. T2DM was 4.24 (95% CI: 3.62, 4.96, *P* < 0.001) but was near unity and nonsignificant when adjusted for the much longer diabetes duration in T1DM than T2DM (odds ratio for type of diabetes 0.97; 95% CI: 0.80, 1.18, *P* = 0.77, on adjustment for duration).

There was no difference in the prevalence of CKD5 by sex in those with T2DM (odds ratio for sex 0.91; 95% CI: 0.80, 1.03, *P* = 0.13). However, for those with T1DM there was a slight preponderance of CKD5 in men: the odds ratio adjusted for age and diabetes duration was 1.25 (95% CI: 1.00, 1.58, *P* = 0.05).

### Point prevalence of RRT

In Scotland, 1.35% of individuals with T1DM and 0.35% of those with T2DM were receiving RRT on 31 May 2008 (Table 1). These figures equate to 83% of those with T1DM and CKD5 and 61% of those with T2DM and CKD5. The difference in rate of RRT by type was not significant (odds ratio for diabetes type 0.83, *P* = 0.43) on adjustment for age, sex and diabetes duration.

**Table 1:** Point prevalence of CKD5 (including RRT) in those with diabetes in Scotland by age and diabetes type

Age group (years)	T1DM			T2DM			Both types		
	Total Number	CKD5		Total number	CKD5		Total number	CKD5	
		Number	%		Number	%		Number	%
≤18	635	4	0.63	8	0	0.00	643	4	0.62
19–35	5585	36	0.64	1064	7	0.66	6649	43	0.65
36–50	7140	141	1.97	16 696	71	0.43	23 836	212	0.89
51–65	4342	108	2.49	56 623	257	0.45	60 965	365	0.60
66–75	1231	21	1.71	52 198	331	0.63	53 429	352	0.66
>75	481	6	1.25	41 282	306	0.74	41 763	312	0.75
Total	19 414	316	1.63	167 871	972	0.58	187 285	1288	0.69

The proportion of those with CKD5 in receipt of RRT decreased by age: 86% of those aged ≤50 years received RRT; 71% of those aged 51–75 years; and 39% of those over 75 years. The odds ratio per year for the trend adjusted for sex, duration and diabetes type was 0.94 (95% CI: 0.93, 0.95, *P* < 0.001).

Of those with CKD5, a greater proportion of men (73%) than women (58%) were receiving RRT (odds ratio for sex adjusted for age, diabetes duration and diabetes type: 1.72; 95% CI: 1.34, 2.21, *P* < 0.001).

### PRDs in those with diabetes receiving RRT

**Figure 2.**
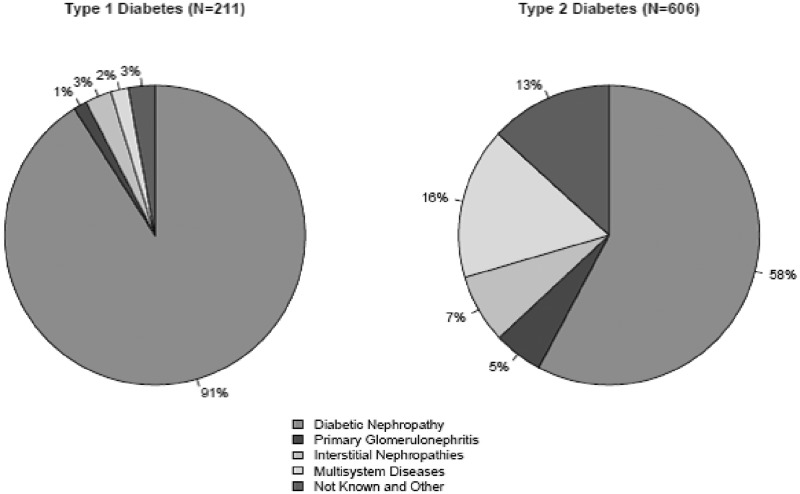
Primary causes of renal disease in those with T1DM and T2DM receiving RRT. Among those with T2DM, multisystem diseases comprise of the following: Renal vascular disease—type unspecified (32%); renal vascular disease due to hypertension (no primary renal disease) (23%); myelomatosis/light chain deposit disease (11%); and renal vascular disease due to other cause (11%).

The designated PRD was unobtainable for 24 (2.9%) people with diabetes receiving RRT (6 patients with T1DM and 79 people with T2DM). A total of 91% of patients with T1DM had a PRD of diabetic nephropathy in contrast to 58% of those with T2DM (*P* < 0.001, Chi-squared test) (Figure 2). Primary glomerulonephritis, interstitial nephritis and multisystem diseases comprised the PRDs for 28% of individuals with T2DM.

### Survival in those with diabetes receiving RRT

The incident cohort for survival analysis comprised 841 people who had first received RRT from 2006 to 2011. Median survival time from initiation of RRT was 3.84 years (95% CI 2.77, 4.62) in T1DM and 2.16 years (95% CI 1.92, 2.38) in T2DM. The unadjusted yearly survival rates following initiation of RRT are shown in Table 2.

**Table 2:** Survival by year from first RRT and type of diabetes

Years since first RRT	Percentage alive (95% CI)		
	T1DM	T2DM	Both types
0	100.00 (100, 100)	100.00 (100, 100)	100.00 (100, 100)
1	83.8 (79.0, 89.0)	71.9 (68.4, 75.5)	75.0 (72.0, 78.0)
2	67.4 (61.0, 74.6)	52.1 (48.1, 56.5)	56.0 (52.5, 59.7)
3	54.8 (47.6, 63.2)	34.4 (30.3, 39.1)	39.6 (35.9, 43.7)
4	47.7 (39.7, 57.2)	24.2 (20.2, 29.0)	30.0 (26.2, 34.3)

Analysis of survival by PRD was restricted to those with T2DM (*n* = 606) given that almost all people with T1DM had a PRD of diabetic nephropathy. Yearly survival for the first 4 years follow-up stratified by PRD is shown in Table 3.

**Table 3:** Survival by year from first RRT with respect to PRD for those with T2DM

Years since first RRT	Percentage alive (95% CI)				
	Diabetic nephropathy	Primary glomerulonephritis	Interstitial nephropathies	Multisystem diseases	Not known and other causes of CKD5
0	100.00 (100, 100)	100.00 (100, 100)	100.00 (100, 100)	100.00 (100, 100)	100.00 (100, 100)
1	74.04 (69.53, 78.84)	84.26 (72.49, 97.93)	79.40 (68.24, 92.38)	59.43 (50.48, 69.97)	70.68 (61.27, 81.54)
2	51.79 (46.42, 57.77)	66.64 (51.53, 86.19)	66.28 (53.22, 82.55)	42.80 (33.88, 54.08)	52.87 (42.49, 65.79)
3	35.65 (30.22, 42.07)	49.98 (32.98, 75.75)	40.03 (26.25, 61.04)	23.05 (15.36, 34.60)	36.00 (25.72, 50.40)
4	24.78 (19.60, 31.34)	44.43 (27.61, 71.49)	30.88 (17.66, 53.99)	17.96 (10.47, 30.82)	17.60 (9.03, 34.32)

### Discussion

This study presents a nationwide analysis of the current burden of CKD5 and RRT in people with T1DM and T2DM in Scotland. We have demonstrated the current prevalence of CKD5 in T1DM and T2DM. Diabetic nephropathy was the PRD in 91% of patients with T1DM but was the PRD in only 58% of patients with T2DM.

Previous studies have tended to report incidence of RRT in diabetes as opposed to prevalence.^12^^,^^13^ A large European study comprising of 10 registries showed a small increase in the incidence of RRT for T1DM and a marked increase in the incidence of RRT in T2DM between 1991 to 2000(4). UK registry data showed 15.2% of prevalent patients had diabetes as their PRD and 25% of new incident patients.^6^ In contrast to these studies, our study determined prevalence of RRT in patients with diabetes and so allows quantification of the current burden of RRT in diabetes on health services thereby allowing future resource planning. It is estimated that by 2030, the prevalence of diabetes will have more than doubled.^14^ A crude estimate quantifying the future burden to the National Health Service in Scotland is that there will be a relative increase in overall national demand for RRT of 22% purely due to increasing diabetes prevalence. This assumes that the current ratio of T1DM to T2DM and prevalence of CKD5 remains stable. This, albeit rough calculation, is a stark indicator of the possible future burden to the National Health Service.

There have been few previous estimates of the incidence or prevalence of CKD5 among diabetic populations.^13^^,^^15^^,^^16^ Other older studies have determined the prevalence of advanced renal disease in subjects with T1DM, using the presence of macroalbuminuria to define advanced renal disease.^17^^,^^18^. Although indicative of advanced renal disease, macroalbuminuria is not specific to, nor required for, a diagnosis of CKD5.^19^ A large global cross-sectional study showed only 24% of those with CKD5 had macroalbuminuria.^20^ Our study differs from the existing literature as the presence of CKD5 is based on eGFR measurements. Our results therefore add reliable, nationwide estimates of the current burden of CKD5 and RRT in people with diabetes to existing knowledge.

We have shown that a greater proportion of prevalent cases of CKD5 with T1DM were in receipt of RRT (83%) than prevalent cases of CKD5 and T2DM (61%) but these differences were accounted for once adjustment was made for age, sex and diabetes duration. Consistent with this, among those with T2DM, people were more likely to receive RRT if they were younger and male.^21–24^

The PRD of patients with T2DM displayed more heterogeneity than in patients with T1DM. This is in keeping with previous biopsy studies that have indicated that T2DM is associated with a greater variety of renal pathology.^25–30^ However, these biopsy series have the inherent potential for selection bias. This study provides valuable evidence of heterogeneity existing on a national scale. Although renal biopsies to determine the PRD in patients with diabetes and CKD are rarely performed if diabetic nephropathy is clinically suspected, the presence of minimal albuminuria, microscopic haematuria, and the absence of diabetic retinopathy, refractory hypertension or rapidly decreasing eGFR are some of the signs that would warrant the consideration of an alternative underlying pathological process and the need for renal biopsy.^19^ The PRD due to causes other than diabetic nephropathy are therefore more frequently biopsy proven and so it is likely that the heterogeneity seen in reported PRDs represents true underlying variety in renal disease pathology. It should be noted that in patients with T2DM there are a greater proportion of missing PRD (11% compared with 3% in T1DM) so that estimates of the prevalence of each PRD are somewhat less certain in those with T2DM than T1DM. Nonetheless the broad picture of greater heterogeneity in type 2 than type 1 is clear.

We have included brief data on survival in patients on RRT to emphasize the ongoing poor prognosis once patients have progressed to needing RRT. In our cohort, the median survival time from initiation of RRT was 3.84 years (95% CI 2.77, 4.62) in T1DM and 2.16 years (95% CI 1.92, 2.38) in T2DM. This, however, is not adjusted for age which would account for this difference. Median survival for the overall population of RRT recipients in Scotland was 4.1 years (95% CI 3.9, 4.2).^31^ As detailed by the UK renal registry report, longer-term survival at younger ages is worse in those with than those without diabetes as a PRD with the differences being less at older ages.^7^

Unfortunately, in our study there were insufficient data to quantify survival rates by individual PRD and to test for significant differences in survival rates by PRD. The observed survival rates in those with diabetic nephropathy were higher than for multisystem diseases but lower than for primary glomerulonephritis and interstitial nephropathy. A larger dataset would be required to define such differences with any precision.

The strength of our study is that it is composed of national renal and diabetes data. We have been able to comprehensively capture people with T1DM and T2DM in Scotland without selection bias creating the largest study of CKD5 in a diabetic population. The prevalence and trends of RRT resulting from diabetic nephropathy as derived from national renal registries can be used as a surrogate marker for ESRD but these data omit those with ESRD who are not receiving RRT. Our study has been able to provide a direct estimate of the current prevalence in all patients with diabetes in Scotland. These findings can be transferred to diabetes patterns in other developed countries.

Limitations of our study include the difficulty when describing the PRD. The physician caring for the patient makes the assessment based on clinical findings and laboratory tests and so this ultimately remains a subjective assessment. The absence of biopsy-proven PRD may give rise to misclassification bias. This method, however, reflects current national clinical practice to which the results are directly applicable. A further limitation of the study is the lack of albuminuria data which are not, as yet, available nationally.

In conclusion, we have provided a current national prevalence of CKD5 and RRT in patients with diabetes. The data are useful in emphasizing the heterogeneity of renal disease in T2DM and provide useful data on renal outcomes among those with diabetes for examining future trends. The prevalence of T2DM is expected to increase worldwide and the incidence of people requiring RRT will also increase dramatically. The increased prevalence will potentially place a significant strain on future healthcare resources. These results, therefore, urge the need for worldwide interventions focusing on the prevention of both diabetes and progression of renal disease as well as allowing future resource planning.
